# Implementation of Stroke Dysphagia Screening in the Emergency Department

**DOI:** 10.1155/2013/304190

**Published:** 2013-02-21

**Authors:** Stephanie K. Daniels, Jane A. Anderson, Nancy J. Petersen

**Affiliations:** ^1^Research Service Line, Department of Communication Sciences and Disorders, Michael E. DeBakey VA Medical Center and University of Houston, 2002 Holcombe Boulevard, Houston, TX 77030, USA; ^2^Health Services Research and Development Center of Excellence, Department of Neurology, Michael E. DeBakey VA Medical Center and Baylor College of Medicine, 2002 Holcombe Boulevard, Houston, TX 77030, USA; ^3^Health Services Research and Development Center of Excellence, Department of Medicine, Michael E. DeBakey VA Medical Center and Baylor College of Medicine, 2002 Holcombe Boulevard, Houston, TX 77030, USA

## Abstract

Early detection of dysphagia is critical in stroke as it improves health care outcomes. Administering a swallowing screening tool (SST) in the emergency department (ED) appears most logical as it is the first point of patient contact. However, feasibility of an ED nurse-administered SST, particularly one involving trial water swallow administration, is unknown. The aims of this pilot study were to (1) implement an SST with a water swallow component in the ED and track nurses' adherence, (2) identify barriers and facilitators to administering the SST through interviews, and (3) develop and implement a process improvement plan to address barriers. Two hundred seventy-eight individuals with stroke symptoms were screened from October 2009 to June 2010. The percentage of patients screened increased from 22.6 in October 2009 to a high of 80.8 in March 2010, followed by a decrease to 61.9% in June (Cochran-Armitage test *z* = −5.1042,  *P* < 0.0001). The odds of being screened were 4.0 times higher after implementation compared to two months before implementation. Results suggest that it is feasible for ED nurses to administer an SST with a water swallow component. Findings should facilitate improved quality of care for patients with suspected stroke and improve multidisciplinary collaboration in swallowing screening.

## 1. Introduction

A well-established best practice in the care of patients with stroke is the early detection of dysphagia as it allows for immediate intervention thereby reducing morbidity, length of stay, and healthcare costs [1–3]. The essential first step to ensure early detection of dysphagia, and to prevent dysphagia-related morbidity, is to screen all stroke patients for signs of swallowing impairment prior to oral intake [1]. When a swallowing screening protocol is implemented, there is a decrease in morbidity over each year that the protocol is in place [4]. Moreover, when hospitals implement a formal swallowing screening protocol for patients with stroke, there is improvement in clinicians' adherence with screening swallowing prior to oral intake [2], and the first dose of aspirin is administered earlier [5]. 

These findings have led the American Heart Association/American Stroke Association (AHA/ASA) to include screening of swallowing prior to the administration of food, liquid, or medication in individuals presenting with stroke symptoms as part of their guidelines on the early management of adults with acute stroke [6]. Within the Veterans Health Administration (VHA) the importance of dysphagia screening in patient with stroke is reflected in the issuance of multiple directives. The Office of the Inspector General (OIG) issued VHA Directive 2006-032 mandating that the initial nurse assessment must include screening of swallowing, and in 2011 the VHA Directive for Treatment of Acute Ischemic Stroke (AIS) required that all VHA facilities include dysphagia screening in their stroke care protocols and track performance as a measure of quality stroke care [7].

Completion of dysphagia screening prior to administration of oral intake was a Joint Commission (JC) required performance measure for Primary Stroke Center Certification until 2010 when it was removed due to a lack of systematically defined standards for what constitutes a valid screening tool for swallowing [8]. The discontinuation by the JC, however, does not indicate that screening swallowing in patients with stroke is no longer a best practice. Rather, it suggests that further research is warranted to obtain consensus on validated swallowing screening tools (SSTs). 

Dysphagia screening protocols for patients with stroke, nevertheless, should include SSTs that incorporate evidence-based swallowing screening items (SSIs). Evidence-based SSIs have been validated for identifying aspiration in patients with stroke based on instrumental evaluation as the reference standard. A process for identifying evidence-based SSIs is described elsewhere [9] and is considered by the authors to be a legitimate interim approach until consensus is reached on specific SSTs or until a VHA-specific stroke SST is identified. Implementation strategies should also include effective training on administration and interpretation of the SST to ensure reliable results and sustainable swallowing screening skills among nurses and other clinicians administering the dysphagia screening protocol. Finally, it is equally important that SSTs are feasible for implementation in various practice settings because even the most valid and reliable SST will not be used if it is not feasible to administer in practice. 

In response to the AHA/ASA guidelines and for compliance with stroke quality performance measures outlined in VHA directives, many VHA facilities have implemented locally developed SSTs for nurses to administer as part of stroke dysphagia screening protocols. There is debate on the need to incorporate trial water swallows with nonswallowing screening items as opposed to using purely nonswallowing screening items in locally developed SSTs. While most SSTs within VHA do not currently include trial swallows [10, 11], water swallows are standard in many SSTs used outside the VHA [12–14]. Furthermore, research provides strong evidence that suggests a water swallow component is critical when screening for dysphagia in individuals with suspected stroke [1, 9]; thus, a water swallow component appears to be an important item to include as part of an evidence-based SST.

Some contend, however, that administration of trial water swallows may compromise patient safety when administered by clinicians without specific expertise in dysphagia screening, such as nurses. There is also the perception that including a trial water swallow component will require extra time to administer thus creating a time constraint that will affect the feasibility of administering the SST. This may be especially true in the emergency department (ED) where there is pressure to complete rapid evaluation and treatment of individuals presenting with suspected stroke. These factors, as well as other unknown barriers, may impede the feasibility of implementing an SST that includes a trial water swallow component.

### 1.1. Purpose of the Study

Since screening of swallowing is a best practice that is essential for safe, high-quality care in individuals presenting with suspected stroke and the VHA has established the OIG and AIS directives, it is paramount that evidence-based mechanisms for screening for dysphagia in veterans with stroke are developed and implemented across VHA. Moreover, a swallowing screening protocol for veterans presenting with symptoms of stroke must be efficient and feasible for use by clinicians in any care delivery setting. The overall objective of this performance improvement study was to identify strategies for effective implementation of swallowing screening in patients with stroke symptoms that presented to the ED at a large VHA facility. 

## 2. Methods

### 2.1. Design

A process improvement approach using a before/after design and qualitative methods was applied to determine the feasibility of implementing an evidence-based SST that included a water swallow component in the ED. The following questions were addressed: (1) Among nurses administering a stroke dysphagia screening protocol in a VHA facility ED, what are the barriers and facilitators to administering an SST with a water swallow component? (2) Does nurses' adherence with screening swallowing prior to oral intake in patients with stroke increase over time after applying process improvement strategies to implement an evidence-based SST with a water swallow component in the ED?

### 2.2. Setting and Sample

The study took place at the Michael E. DeBakey Veterans Affairs Medical Center (MEDVAMC) located in Houston, TX. The MEDVAMC is certified by the JC as a Primary Stroke Center and has the largest number of stroke admissions within the VHA. The ED is staffed with 20 registered nurses (RNs) and 3 emergency medicine physicians. A convenience sample of ED nurses (*N* = 8) was recruited to participate in semistructured interviews to obtain feedback on barriers and facilitators to implementing an SST with water swallow in the ED. Participants were recruited via personal invitation and email solicitation, and they all provided written consent prior to participation. The study was approved by the Institutional Review Board at Baylor College of Medicine and by the Research and Development Committee at the MEDVAMC.

### 2.3. Planning and Assessing the Implementation

In meeting study objectives, Plan, Do, Study, Act (PDSA) cycles were applied to identify process improvement strategies for implementation of an evidence-based SST with water swallow in the ED. The PDSA cycle is a well-established process improvement methodology that can be used to implement quality improvement changes in the “real-world” practice setting [15]. Prior to establishing an evidence-based nurse-administered stroke SST, swallowing screening for stroke patients was conducted in a nonstandardized fashion primarily by ED physicians and neurology residents, and infrequently by nurses.

The first step, “Plan” was accomplished by a multidisciplinary team of speech pathologists and nurses with expertise in stroke and dysphagia. From June–September 2009, the team developed an evidence-based stroke SST that included a water swallow component. Items incorporated in the stroke SST were based on literature review [16–19] and expert consensus agreement (Table 1). A stroke dysphagia screening protocol was then developed to guide administration of the SST and clinical interventions based on screening results. The protocol required that nonswallowing “observational” items be administered first. If any nonswallowing item was evident, the screening was discontinued. The patient continued nil per os (NPO), that is, nothing by mouth including medication, and speech pathology was consulted. If none of the observational items were present, trial water swallows were initiated starting with a 5 mL volume. Each volume was administered twice. If cough, throat clear (audible attempt to clear material out of the throat), or wet voice was evident after any water trial, the screening was stopped and no further water was administered. The patient continued NPO status, and speech pathology was consulted. If cough, throat clear, or wet voice was not evident, the patient was considered to have no risk of dysphagia and oral intake was initiated.

The second step, “Do” involved implementing the swallowing screening protocol in the ED at the MEDVAMC. Implementation strategies began with initial education sessions from December 2009 to mid-January 2010 for all ED nurses in which information was presented on (1) the current guideline-derived best practices for swallowing screening in patients with stroke [6, 7], (2) how to administer the stroke SST with water swallow [16], and (3) specific protocol actions required based on whether the patient passed or failed the SST [6, 7]. After training sessions were completed, the ED nurses implemented the SST with a water swallow component as part of the stroke dysphagia screening protocol starting in December 2009. 

The third step, “Study” involved tracking of nurses' adherence to the swallowing-screening protocol as it was implemented over time and also conducting semistructured interviews with ED nurses to identify barriers and facilitators encountered during implementation of the swallowing-screening protocol. Semistructured interviews were conducted in March 2009 and were designed to elicit feedback from the nurses responsible for administering the SST with water swallow. The interviews lasted 20 minutes and were audio recorded for transcription. Participants were asked to describe their experience in administering the SST including barriers and facilitators to completing the screening including the water swallow section, what they liked and disliked about the SST, and what facilitated and impeded documentation in the electronic health record (EHR) and to provide ideas on how to make the process of administering the SST better for ED nurses. Audiotapes from the interview sessions were transcribed and coded using content analysis. Words and word phrases were categorized as being indicative of either a barrier or a facilitator to administering the SST [20]. From March to April, minimal contact with the ED nurses was made as data were analyzed; however, SST implementation continued.

The final step, “Act” was initiated in April 2009 and included implementing multiple strategies and lessons learned based on feedback from ED nursing staff. This involved the application of rapid PDSA cycles [15] to target identified barriers and was an iterative process completed over a 3-month period. One important product was the development of a Stroke Dysphagia Screening Bundle (SDSB) that included (1) an evidence-based SST with water swallow, (2) EHR order sets that automated NPO status and consultation to speech pathology for patients with a positive SST and diet orders for patients with a negative SST, and (3) electronic templates that automated documentation of the entire dysphagia screening process in the EHR. 

To address implementation barriers, the same process of PDSA cycles was applied, and implementation methods and education modules were developed and tailored to address the needs of the nurses administering the SDSB. Implementation tools and education modules were made accessible via a web interface for easy access and for booster training as needed. 

### 2.4. Data Analysis of Pre-/Postimplementation Measures

Adherence with implementing an evidence-based SST with water swallow in the ED was assessed before and after implementation of the SDSB. This was accomplished by reviewing the EHRs of patients admitted to MEDVAMC with stroke symptoms and tracking if these patients received screening of swallowing prior to oral intake. EHRs were reviewed from October and November 2009 (the two months prior to implementing the SDSB) with continuation to June 2010 to identify the percent of patients screened each month in the ED post-SDSB implementation. The Cochrane-Armitage test was used to test if there was a trend in the percent of patients screened over the 9 months. Logistic regression was used to calculate the odds of being screened in the 7 months following implementation compared to the 2 months before implementation.

## 3. Results

**Figure 1:**
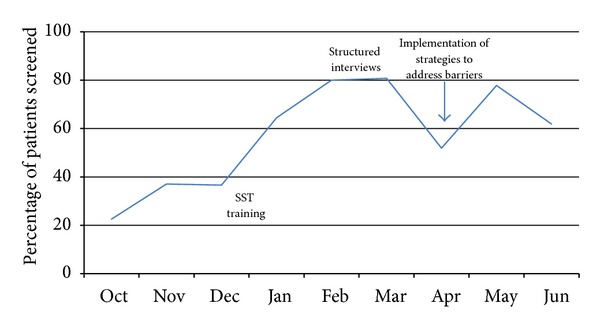
Percentage of patients screened in the emergency department from October 2009 to June 2010.

A total of 278 individuals with stroke symptoms were screened in the ED from October 2009 to June 2010. The percentage of patients screened increased from 22.6% in October 2009 to a high of 80.8% in March 2010, followed by a decrease to 61.9% in June 2010. Following implementation of the SST, the percentage of patients screened decreased to its lowest point of 51.9% in April 2010 but rebounded to 77.8% in the following month after implementing strategies to address identified barriers. There was a significant increase in the percentage of patients screened in the ED over time (Cochran-Armitage test *z* = −5.1042,  *P* < 0.0001) (Figure 1). The odds of being screened was 4.0 times higher after implementation (95% CI, 2.2 to 7.3), compared to the 2 months before implementation.

 Barriers identified from nurses' interview sessions were (1) difficulty finding time to document screening results in the EHR, (2) difficulty recalling all screening items during administration of the SST, (3) inconsistent administration of the SST, and (4) inaccurate interpretation of screening items, (e.g., confusing the item somnolence with the assessment of a patient's level of orientation or administering a patient 5 mL of water using a syringe instead of having the patient drink water from a cup). 

 Key facilitator themes that were subsequently applied in developing implementation strategies were (1) more education on dysphagia and evidence-based screening of swallowing, (2) efficient processes to support SST administration and interpretation, and (3) multidisciplinary team cooperation and support from ED administrators. The time it took to administer the SST was not formally recorded. However, during interview sessions, nurses reported, on average, the SST took approximately 5 minutes. Interestingly, no nurse reported that administration of the water swallow component was a barrier to completing the SST.

**Figure 2:**
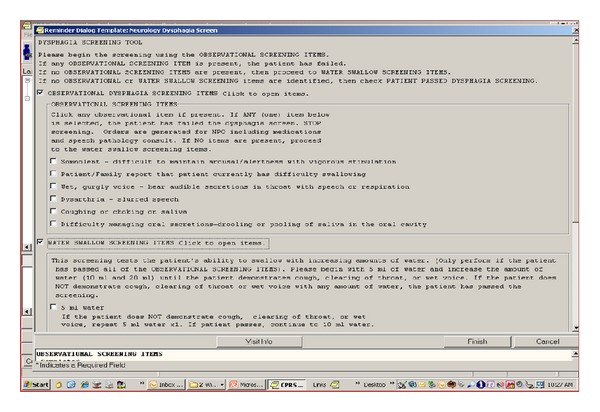
Template of swallowing screening in the electronic health record system.

 To facilitate the incorporation of the SDSB into daily practice, implementation methods and education materials were tailored to address identified barriers. Pocket cards were provided as a reminder aid (e.g., listed all SST items and steps for administration), and electronic tools (order sets and templates) were developed in the EHR to automate the steps of the SDSB and to facilitate documentation of SST results (Figure 2). An online video training module was produced to illustrate appropriate administration and interpretation of the SST, and booster education sessions were tailored to the specific needs of the nurse and targeted areas of identified deficit.

## 4. Discussion

This current performance improvement study was designed to determine the feasibility of implementing a nurse-administered stroke SST with a water swallow component and to identify strategies for effective implementation of a dysphagia-screening protocol for patients with stroke symptoms who present to the ED. We are unaware of any previous research that has assessed the feasibility of a nurse-administered SST, particularly an SST for use in the ED. The three major findings of this implementation study were as follows (1) an SST with a water swallow component was feasible for nurses to complete in patients presenting to the ED with symptoms of stroke; (2) an SDSB was created and swallowing screening significantly improved over time after implementation; (3) tailored implementation and education methods with booster sessions improved the sustainability of nurses' adherence with implementing the SDSB. Thus, the bundling of evidence-based SST items, swallowing screening processes, and clinical interventions with tailored implementation and education methods significantly improved stroke dysphagia screening at our facility.

 Screening swallowing prior to oral intake in individuals presenting to the hospital with stroke symptoms is an important best practice described in AHA/ASA guidelines [6]. Since many patients with stroke symptoms first present to the ED, screening swallowing in the ED is most appropriate. This is based on the rationale that screening swallowing early, at the first point of patient contact, has the greatest potential to prevent administration of oral intake or oral medications prior to completing dysphagia screening. It is not uncommon for stroke patients in the ED to require immediate interventions to control blood pressure, discomfort, and other medical concerns that are often treated with oral medications. The ED, however, is extremely busy, with nurses responsible for multiple care processes in the stroke work-up, and completing an SST that includes a water swallow component will add to nurses' responsibilities. Yet, all nurses in this study welcomed the opportunity to objectively determine the feasibility of implementing an SST with water swallow and to identify strategies for effective stroke dysphagia screening in the ED. 

### 4.1. Focused Implementation Strategies

We engaged nursing staff in identifying facilitators and barriers for administration of an SST with water swallow in the ED and sought their input in developing strategies to address identified implementation barriers. Two implementation strategies emerged: (1) “bundling” dysphagia screening processes and (2) “tailoring” implementation and education methods. The multiple sequential actions involved in administering the SST and the required clinical interventions were bundled into an all inclusive SDSB. 

Bundles are defined as a set of evidence-based interventions for a specific patient population and setting that when implemented together result in significantly better outcomes than when implemented individually [21]. The Institute for Healthcare Improvement and other groups recommend the use of “care bundles” to improve patient care and clinical outcomes [22]. Bundles have been most effective in improving the quality of care for mechanically ventilated patients by improving healthcare providers' compliance with relevant evidence-based practices. Bundles have also been shown to improve effective assessment of pain, appropriate use of blood transfusions, appropriate sedation, appropriate peptic ulcer prevention, and appropriate deep vein thrombosis prophylaxis [21]. 

 The application of the bundle concept to improve dysphagia screening in patients with stroke is well suited because the primary purpose of a bundle is to pull together the essential evidence-based interventions (SST and associated clinical actions) that target a specific patient population (patients with stroke) undergoing a particular procedure (dysphagia screening) to ensure the best possible patient care and outcomes (prevent dysphagia-associated morbidity/mortality). The essential evidence-based interventions needed to develop an SDSB are as follows: (1) maintain the patient on a NPO status (including medications) until administration and interpretation of an evidence-based SST [6], (2) administer an evidence-based SST and interpret findings, (3) initiate oral diet without dysphagia modifications and initiate oral medications if SST results are negative, (4) continue NPO status and consult speech pathology to complete a swallowing assessment if screening results are positive, and (5) document completion of each SDSB component in the EHR. 

 Tailoring involves adapting or modifying interventions, implementation strategies, and educational resources to fit a specific population or context. Tailoring appears to be a critical factor related to effective implementation and is associated with improvements in process and patient outcomes [23, 24]. Effective tailoring requires engagement of stakeholders in developing implementation strategies to address identified barriers. For example, when the nurses in this study reported it was difficult to remember each screening item and the specific steps for the water swallow component included in the SST, they suggested developing a pocket card that listed each screening item with instruction on how to administer the water swallow component. Tailoring supports the unique needs of health care providers delivering the intervention but also makes possible adjustments in the intervention based on specific needs [23, 24]. Consistent administration of the SST and documentation of the administration process and findings in the EHR were an identified barrier. Nurses recommended developing an electronic template in the EHR that provided the step-by-step process used to administer the SST and would simultaneously document each step of the SST that was completed as the nurse interacted with the template. Later in the implementation process when the SDSB was developed, automated data sets were also incorporated into the EHR. 

 In terms of education, tailored strategies included providing ongoing training to nursing staff when deficits were identified. Performance monitoring and feedback that included the percentage of SSTs completed each month was reported to nursing staff in the ED and was used as an incentive and as an indicator of learning need. The investigators produced a video-training module of the SST procedure and made it accessible to all nursing staff on a facility intranet site. This provided easy access for booster training sessions when performance monitoring showed decreases in swallowing screening rates or when learning needs were identified by nursing staff. 

### 4.2. Sustainability

The MEDVAMC receives the largest number of admissions for suspected stroke compared to other VHA facilities. In Fiscal Year 2009, approximately 20 patients with stroke symptoms were admitted monthly. Even with this high volume, an individual nurse may have the opportunity to complete an SST only once a month, so maintaining consistent and reliable administration and interpretation of the SST is challenging and may affect sustainability as evidenced by our fluctuating numbers in screenings during the study period. Consistently high screening numbers were observed following initiation of the screening protocol when education was fresh and the initiative was a new endeavor and again when nurses were engaged in developing strategies to address identified implementation barriers. 

 This finding is in line with much of the literature on the adoption of innovation which claims that adoption of a best practice or innovation is not a one-time occurrence, but rather a complex process that develops over time [25]. Adoption can be described in three stages: preadoption, early use, and established use [26]. The first stage, preadoption, occurs early on with the first introduction to a new innovation. Adoption of the innovation builds as the intended adopters have sufficient knowledge about what the innovation is, how it is used, and how it benefits them. The next stage, early use, includes periods of fluctuation as adoption builds to more consistent use. This occurs when adopters are provided continued access to information about the innovation and receive sufficient training to support innovation tasks. During the final phase, established use becomes more evident and is related to adopters receiving adequate feedback on performance and sufficient opportunity to adapt and refine the innovation to improve its fit based on setting and context. 

 The fluctuation seen in the adoption of the SST by ED nurses in this study is most indicative of the stages of preadoption and early use. During the first few months of this 9-month pilot study, nurses were first introduced to the SST and received ongoing information about the SST, training on how to use it, and opportunities to adapt and develop implementation strategies. During the final 3 months of the project, targeted strategies developed by the ED nurses were initiated. 

Another trend that may be attributed to the fluctuation in swallowing screening rates is that increases in swallowing screening were observed after periods of engagement with ED nurses (i.e., training and interview sessions) and decreases were observed in swallowing screening during times of minimal engagement with the ED staff. This finding strongly suggests that periodic engagement of the ED staff and availability of educators to answer questions and to provide ongoing booster sessions are important for sustained adoption. Moreover the involvement of the ED staff throughout the implementation process appeared critical to success and sustainability. Although fluctuation in screening adoption was evident, it is important to note that screening adherence never dropped to preimplementation levels further supporting the implementation of an ED nurse-administered SST with a water swallow component.

 Since completion of this pilot study, we have continued to track performance with dysphagia screening and provide performance feedback to ED staff. The SDSB has been adopted at the MEDVAMC to implement evidence-based dysphagia screening and now includes ongoing booster education when deficiencies in dysphagia screening are identified. To date, the SDSB has been effective for consistent implementation of evidence-based dysphagia screening for patients admitted with suspected stroke with sustained screening rates between 93% and 100%. These data support adoption of the SDSB at the stage of established use. Subsequent steps are to test the effectiveness of developing site-specific SDSBs and tailored implementation methods and education in multiple VHA facilities.

### 4.3. Limitations

The time period evaluated was approximately 9 months. Continued longitudinal research is warranted to determine if results are maintained. Furthermore, implementation of this procedure should be completed at large and small medical centers to determine if the implementation process and results are similar. The time to administer any SST is an important focus of future research to ensure feasibility. 

 Every ED may not be able to complete a stroke SST given high-volume patient load, rapid transfer to patient wards, and/or limited staff. In cases where screening cannot be completed in the ED, it is unclear if this implementation process would work on a hospital ward and requires further research.

 While the SST developed for the MEDVAMC is evidence based, it has not been validated. Work is in progress to develop and validate a VHA stroke SST in a separate study. Since several VHA directives have charged VHA facilities with implementing and monitoring dysphagia screening protocols for patients with stroke, evidence-based SSTs and effective implementation strategies are needed now. Thus, implementation studies should be completed in parallel with validation studies and once an SST is validated, effective implementation processes will be in place.

## 5. Conclusions

It is well established that patient outcomes are improved when dysphagia screening is completed prior to oral intake in individuals with stroke symptoms. This body of work supports the feasibility of nurses screening swallowing using an SST with water swallow in patients with stroke symptoms that present to a busy ED. Engaging nursing staff in the process of identifying barriers and targeted solutions resulted in the development of an SDSB and tailored implementation and education methods that significantly improve dysphagia screening adherence over time. Continued interaction and booster education sessions on administering and interpreting the SST are required for sustained improvement and consistent practice. 

## Figures and Tables

**Table 1:** Michael E. DeBakey Veterans Affairs Medical Center stroke swallowing screening tool.

Non-swallowing items	
Somnolent-difficult to maintain arousal/alertness with vigorous stimulation	
Wet, gurgly voice quality-hear audible secretions in the throat with speech or respiration	
Dysarthria-slurred speech	
Drooling or pooling of saliva in oral cavity-difficulty managing saliva in the mouth	
Coughing, choking on saliva	
Patient/family reports patient with current difficulty swallowing	
Swallowing items	
5 mL water ×2	
10 mL water ×2	
20 mL water ×2	
